# gapTrick—structural characterization of protein–protein interactions using AlphaFold

**DOI:** 10.1093/bioinformatics/btaf532

**Published:** 2025-09-23

**Authors:** Grzegorz Chojnowski

**Affiliations:** European Molecular Biology Laboratory, Hamburg Unit, Hamburg 22607, Germany

## Abstract

**Motivation:**

The structural characterization of protein–protein interactions is a key step in understanding the functions of living cells. Here, I show that AlphaFold3 often fails to predict protein complexes that are either weak or dependent on the presence of a cofactor that is not included in a prediction.

**Results:**

To address this problem, I developed gapTrick, an AlphaFold2-based approach that uses multimeric templates to improve prediction reliability. I demonstrate that gapTrick improves predictions of weak and incomplete complexes based on low-accuracy templates, such as individual protein models that have been rigid-body fitted into cryo-EM reconstructions. I also show that gapTrick identifies residue–residue interactions with high precision. These interaction predictions are a very strong indicator of model correctness. The approach can aid in the interpretation of challenging experimental structures and the computational identification of protein–protein interactions.

**Availability and implementation:**

The gapTrick source code is available at https://github.com/gchojnowski/gapTrick and requires only a standard AlphaFold2 installation to run. The repository also provides a Colab notebook that can be used to run gapTrick without installing it on the user’s computer.

## 1 Introduction

Structural characterization of protein–protein interactions (PPIs) is essential for elucidating roles of protein-coding genes in biological pathways. It is critical for understanding protein interaction mechanisms, regulatory roles of post-translational modifications (PTMs), and disruptive effects of disease mutations. Experimental approaches like pull-down assays and cross-linking mass spectrometry (XL-MS) can provide some insight into PPI patterns but are difficult to apply for large-scale analyses and often produce inconclusive results (Kosinski *et al.* 2015).

Macromolecular crystallography (MX) provides one of the most detailed experimental information on protein structures and was used to determine the majority of structural models available in Protein Data Bank (PDB) (The wwPDB Consortium 2019). While the crystallization of small molecules tends to result in optimal packing, macromolecules crystallize in space groups that promote connectivity, preserving internal symmetry of stable complexes (Wukovitz and Yeates 1995). Therefore, crystallization can be seen as a natural docking experiment in which multiple alternative interactions between macromolecules form a crystal lattice (Krissinel 2010). In practice, however, separation of stable, biologically relevant interactions from crystal contacts is not trivial (Krissinel and Henrick 2007).

Cryo-EM models are generally easier to interpret because they correspond to complexes that are stable in solution, but similarly to MX, they frequently represent small parts of functional complexes in a way that hinders identification of all interactions relevant to their function (Poweleit *et al.* 2019). Moreover, cryo-EM models often lack the details needed to reliably identify interactions such as hydrogen bonds or salt bridges, which is crucial for their interpretation and experimental validation. As of January 2025, 40% of the EM reconstructions deposited to EMDB (Turner *et al*. 2024) were determined at a reported resolution below 4 Å, meaning that half of their voxels have local resolution significantly worse than 4 Å and do not allow for an accurate model building (Chojnowski *et al.* 2021).

Artificial intelligence (AI)-based protein structure prediction methods have quickly become an important alternative to the experimental identification of PPIs (Molodenskiy *et al.* 2025). However, they also proved to be computationally expensive and have limited sensitivity. For example, in a recent study targeting binary interactions in a human proteome only 5% of expected interactions were recovered with high confidence (Burke *et al.* 2023).

The main limitation of the AI-based tools such as AlphaFold2 (AF2) (Jumper *et al.* 2021) is the limited sampling of conformational space near the native state, not their ability to reliably identify a correct prediction (Roney and Ovchinnikov 2022). As a result prediction accuracy of poorly conserved monomeric proteins, with shallow MSAs, strongly depends on the availability of homologous template structures (Jumper *et al.* 2021, Tunyasuvunakool *et al.* 2021). The contact information derived from templates complements weak evolutionary covariation signals that could be used to identify intramolecular interactions defining a native state of a protein. Although not very well studied, this effect can be even more pronounced for multimeric proteins. Strong protein–protein binding interfaces tend to be highly optimized, involving many interactions that result in a higher evolutionary pressure and sequence conservation (Tokuriki and Tawfik 2009). This may allow for more reliable contact identification and prediction of a native-like structure. Weaker complexes that are less dependent on specific interactions would be more difficult to predict with methods like AlphaFold2. The use of multimeric templates could complement the missing evolutionary information, but it has been deliberately blocked in all AlphaFold versions by masking inter-chain interactions from input templates. Presumably, this was done to avoid potential input bias from low-quality templates.

It has been shown that a modification of AlphaFold2, nicknamed AF_unmasked, which uses interchain contacts from input templates, allows predictions of native structures of difficult protein–protein complexes (Mirabello *et al.* 2024). However, this approach has been tested on a relatively small set of 251 heterodimeric complexes released after training the AF2-Multimer (Evans *et al.* 2021) neural network (NN) models, but not otherwise checked for overlap with the training set (Bernett *et al.* 2024). The method can also be computationally expensive in difficult cases.

Here, I present gapTrick, an approach that achieves results comparable to AF_unmasked at an order of magnitude lower computational cost. I show that gapTrick can successfully predict PPI structures that cannot be predicted by standard approaches when a suitable multimeric template is given as input. It uses AlphaFold2 NN models trained on monomeric structures allowing validation using any known structure of protein–protein complexes, as none of them have been used for training. Here, I have built a benchmark set of 3978 stable homo- and hetero-dimeric, trimeric, and tetrameric protein complexes derived from PDB-deposited crystal structures using PISA software (Krissinel and Henrick 2007). I show that gapTrick can complement AlphaFold3 (AF3) (Abramson *et al.* 2024) in predicting weak complexes when a template is available, e.g. from a rigid body fit of complex components in a cryo-EM map. I also show that the approach can reliably predict inter-chain interactions in protein–protein complexes. This can help in the interpretation of medium and low-resolution MX and cryo-EM models that do not allow for a direct identification of residue–residue interactions. The predicted contacts are also a very strong indicator of the model correctness. The approach can help to rebuild experimental models observed in a conformation different from that predicted by AlphaFold2, which otherwise would require tedious rebuilding using interactive software.

The method is nicknamed the ‘gapTrick’ after the original ‘trick’ of combining multiple protein sequences, interspersed with ‘gaps’, into a single chain to enable prediction of protein complexes with the monomeric AlphaFold2 NN model. It has been discussed on social media (Evans *et al.* 2021) and implemented by several groups prior to the release of AlphaFold2-multimer (Bryant *et al.* 2022, Mirdita *et al.* 2022, Humphreys *et al.* 2024). None of these implementations combine the trick with the use of multimeric templates.

## 2 Materials and methods

For simplicity, I use the following assumptions and definitions throughout the paper:

- Two residues are in contact if the distance between their Cβ atoms (Cα for glycine) is <8 Å. This arbitrary cut-off distance does not imply a physical interaction (e.g. a hydrogen bond formation).
- The gapTrick contact predictions are based on AlphaFold2 distograms (Jumper *et al.* 2021). Here, only inter-chain contacts with a probability greater than an arbitrary threshold of 0.8 are analysed.
- All gapTrick benchmarks are based on templates generated from reference models with each chain rotated and translated by random angles and distances, as described in Materials and methods.

### 2.1 Description of the gapTrick algorithm

The prediction of protein–protein complexes in gapTrick is based on AlphaFold2 NN models trained on single-chain proteins. The method uses only two out of five AF2 NN models that allow for the use of input templates (1 and 3). Therefore, by default, only two structural models are generated for each target, compared to 25 in AlphaFold-Multimer and AF_unmasked (5 predictions for each of the 5 NN models). To allow prediction of multimeric structural models, all input protein chains are merged into a single protein chain interspersed with 200 amino acid gaps. The only input required from user is a PDB/mmCIF template and a FASTA file containing all target sequences in arbitrary order. The structure prediction is performed fully automatically in the following steps:

1. Multiple Sequence Alignments (MSAs) are generated separately for all input chains. This step uses the MMseqs2 API (Steinegger and Söding 2017) by default, but the alignments can be also provided by the user in a3m format.
2. Target sequences are assigned to the template chains using a greedy algorithm maximising sequence identity
3. Following the sequence assignment, the template chains and target sequences are merged into single protein chains with residue index gaps (200 by default)
4. MSAs for successive target sequences are merged into a single MSA. No sequence pairing is used at this step, as it is not required for successful prediction of protein complexes (Gao *et al.* 2022)
5. The resulting single-chain protein structure is predicted using a standard AlphaFold2 pipeline with default parameters
6. The predicted models are broken down into multiple chains and ranked by the pTM score (ipTM score is not predicted by monomeric AF2 NN models). Top-ranked model is subjected to the AMBER force field relaxation.

### 2.2 Benchmark set of MX structures from PDB

A benchmark set of stable in-solution protein complex structures was generated using PISA software based on PDB-deposited protein-only crystal structures. Since the reliability of the PISA analysis depends heavily on the quality of the model coordinates (Krissinel 2010), for the analysis, I selected crystal structure models deposited with experimental data, solved at a resolution of 2.5 Å or better and with *R*_free_ <0.25. For computational efficiency of model-prediction, I additionally restricted the set to structures with molecular weight between 20 and 50 kDa. As of 23 September 2024, this procedure resulted in 13 539 protein crystal structure models that were subsequently processed by PISA to identify stable dimeric, trimeric, and tetrameric protein complexes (both homo and hetero oligomers were accepted). From each crystal structure model, only a single top-ranked complex of a desired multimeric state with all protein chains longer than 50 amino acids was selected. The selection procedure ensures that no more stable complexes with the same stoichiometry can be found within the crystal structure. All selected complexes have a non-negative Gibbs free dissociation energy (Δ*G*_diss_). However, after removing non-protein components from the structures (ions, waters, and small molecules), the energy estimate for some of the complexes became negative, suggesting that the presence of the removed components may be critical for their stability. These structures have been retained in the benchmark set as they reflect realistic scenarios for modelling complexes before components critical to their stability have been characterized. The final benchmark set contains 3978 structural models of protein–protein complexes, including 2000 dimers, 722 trimers, and 1256 tetramers.

To mimic low-accuracy experimental models (e.g. rigid-body fitted in cryo-EM maps), templates for the use with gapTrick were generated from reference models by rotating and translating all chains about their centres of mass by uniformly distributed random values up to 30 degrees and 5 Å. This procedure is hard-coded into gapTrick and can be invoked with the - -*rotrans = 30,5* keyword.

It is not a purpose of this work to systematically benchmark AlphaFold3, but to show that its performance may be improved for some cases, using additional data. Therefore, the benchmark set used here completely neglects any potential overlap with the AF3 training set.

### 2.3 Benchmark set of putative binary complexes

From the PPIs defined by hu. MAP 2.0 database (Drew *et al.* 2021), a positive and negative sets of 300 binary complexes each were randomly selected and their structures predicted using AlphaFold3 with default parameters.

Experimentally characterized binary complexes were extracted from PDB-deposited cryo-EM structures determined at resolutions between 4 and 10 Å using PISA. From all potential complexes identified by the program, only those with interface hydrophobicity *P*-value below 0.5 were selected. As of 15 February 2025, 320 models of binary complexes were extracted using these criteria.

### 2.4 Numerical methods

Correlation coefficients shown in the results are bootstrap estimates with 100 repeats. All the statistical analyses were performed using tools implemented in Scipy (Virtanen *et al.* 2020) and Numpy (Oliphant 2006). Figures were prepared using matplotlib (Hunter 2007) and ChimeraX (Pettersen *et al.* 2021). AlphaFold3 predictions were obtained with default parameters using a standalone installation of a version 3.0.0 and weights downloaded on 6 December 2024. The gapTrick code is based on AlphaFold version 2.3.2 and uses AF2 NN models dated 14 July 2021. The cryo-EM model was built and refined using CCP-EM v2 (Burnley *et al.* 2017), MOLREP version 11.9.020.9 (Vagin and Teplyakov 2010), Servalcat version 0.4.32 (Yamashita *et al.* 2021), and COOT version 0.9.8.93 (Casañal *et al.* 2020). For model validation and analysis, I used MolProbity (Prisant *et al.* 2020, Sobolev *et al.* 2020) and PISA distributed with CCP4 version 8.0.018 (Agirre *et al.* 2023). Molecular models processing was performed using scripts based on Gemmi (Wojdyr 2022), CCTBX (Grosse-Kunstleve *et al.* 2002), and BioPython (Cock *et al.* 2009). Multiple sequence alignments used for benchmarks were prepared using a standalone version of MMseqs2 release 14 (Steinegger and Söding 2017) and a colabfold_search script (Mirdita *et al.* 2022). The gapTrick release by default uses MMseqs2 API. Both standalone MMseqs2 installation and API were invoked with - -*use-env 0* and - -*filter 0* options. The TM-scores of predicted structures to the reference models were calculated using Foldseek release 9 (Van Kempen *et al.* 2024) using the easy-multimersearch option.

## 3 Results

### 3.1 The accurate prediction of weak complexes often requires a template

The PISA software performs an exhaustive analysis of inter-chain contacts in crystal structures to identify subsets of interacting molecules that are potentially stable in solution and may have biological relevance. PISA estimates Gibbs free dissociation energy of a complex (Δ*G*_diss_) using chemical thermodynamics approximations based on the interface hydrophobicity and specific interactions like hydrogen bonds, salt bridges, disulfide, and covalent bonds. In principle, all complexes with Δ*G*_diss_>0 are stable in solution, but the prediction accuracy increases with the binding energy; it is very high for strong binders but can be overestimated for weaker ones. It has been shown that as a rule of thumb complexes for which the dissociation energy of protein-only components exceeds 40 kcal/mol are stable in over 99% of cases (Krissinel 2010).

**Figure 1.**
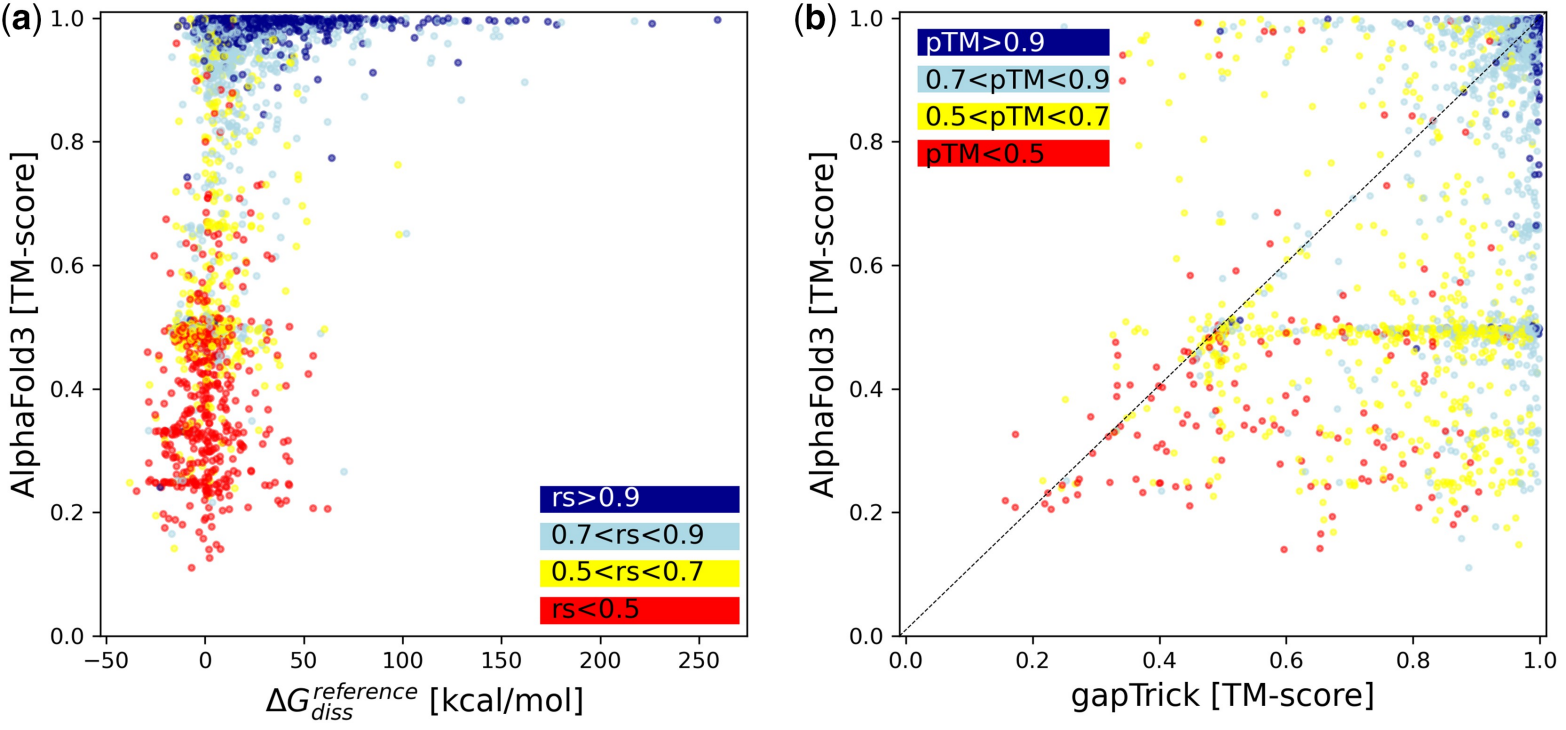
A comparison of the performance of AlphaFold3 and gapTrick for a benchmark set of dimeric, trimeric, and tetrameric protein complex targets, which were predicted by PISA to be stable in solution. The Gibbs free dissociation energy (Δ$G_{\text{diss}}^{\text{reference}}$) was estimated for the reference complexes after removal of all non-protein components. The accuracy of the AlphaFold3 models is significantly reduced for weak protein complexes (a) but can be largely compensated using multimeric templates (b). The colour code indicates the ranking score (rs) of AlphaFold3 (a) and the pTM of gapTrick predictions (b).

I observed that after removing all non-protein components from the benchmark set structures, the dissociation energy estimate of some of them became negative, clearly showing that their stability depends on the presence of additional components (Fig. 1a). AlphaFold3 fails to predict the structure of 38% of complexes with dissociation energy below 50 kcal/mol (TM-score < 0.8), but correctly assigns most of them low ranking scores (Abramson *et al.* 2024) below 0.5. The structure of most of these complexes can be correctly predicted with gapTrick if a template (reference model with randomly shifted and rotated complex components) is given on input (Fig. 1b). The use of multimeric templates increases the fraction of correct predictions (TM-score > 0.8) for targets with dissociation energy below 50 kcal/mol from 62% to 82%.

### 3.2 gapTrick PPI interface contact predictions are highly specific

Reliable identification of residue–residue interactions in protein complexes is important for understanding their function. It is also a key step of the model validation, which typically relies on structure-driven identification of interactions that are critical for their function *in vivo*. This can be challenging for models, either predicted or experimental, that lack fine structural detail. A natural application for the gapTrick would be rebuilding low-quality, initial models, e.g. rigid-body fitted into cryo-EM maps, to identify crucial interactions and help in model interpretation.

**Figure 2.**
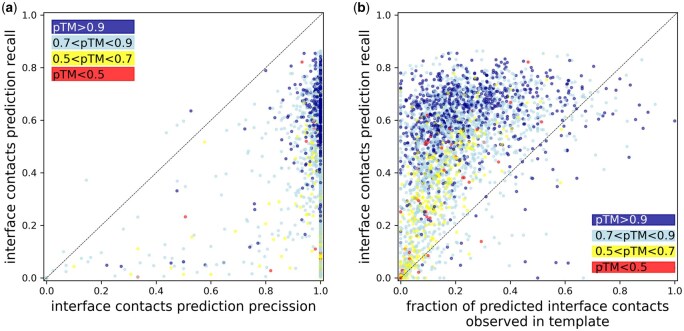
Reliability of the protein–protein interface contacts predicted by gapTrick. The figure shows the recall of inter-chain (interface) contact predictions as a function of contact prediction precision (a) and the fraction of predicted contacts that are satisfied in a template (b). The colour code indicates the pTM score of the gapTrick predictions.

Benchmark results show that contacts identified using gapTrick on protein complex interfaces are very precise, even in low recall cases when only a fraction of expected contacts is predicted (Fig. 2a). Given the high precision of the contact predictions, a good template, close to a reference structure, will have many contacts that are also predicted by gapTrick with high probability. Indeed, the contact prediction recall depends heavily on the fraction of predicted contacts that are also observed in a template (Fig. 2b).

Contact prediction recall for very high confidence models (pTM > 0.9) is weakly dependent on the fraction of predicted contacts observed in a template (Spearman’s *r* = 0.34 ± 0.04), implying that they rely largely on information available in MSAs (Fig. 2b). However, the recall of interface contact predictions in lower confidence models (pTM < 0.9) is significantly reduced for poor templates (Spearman’s *r* = 0.84 ± 0.01), indicating their important role in complementing the weak evolutionary information available in MSAs. The observed high precision of contact predictions agrees with an previous results showing that predictions are generally more reliable than experimental coordinates, even though the underlying AlphaFold2 models were trained on them (Sánchez Rodríguez *et al.* 2024).

### 3.3 gapTrick predicts reliable PPI interface coordinates

Although gapTrick predictions provide reliable information on potential residue-level contacts at the complex interfaces, it is not clear how reliable the model coordinates are at the PPI interface, which may be important for model interpretation. To test this, I compared the PISA software estimates of Gibbs free energy of dissociation (Δ*G*_diss_) for gapTrick predictions and corresponding reference models. PISA is commonly used as a reference for oligomeric-state estimates in PDB-deposited crystal structure models. The program, however, is known to strongly rely on the accuracy of atomic coordinates of the input models, which is required to correctly estimate specific interactions between interface-residues; hydrogen bonds, salt-bridges, and disulfide bonds (Krissinel 2010).

**Figure 3.**
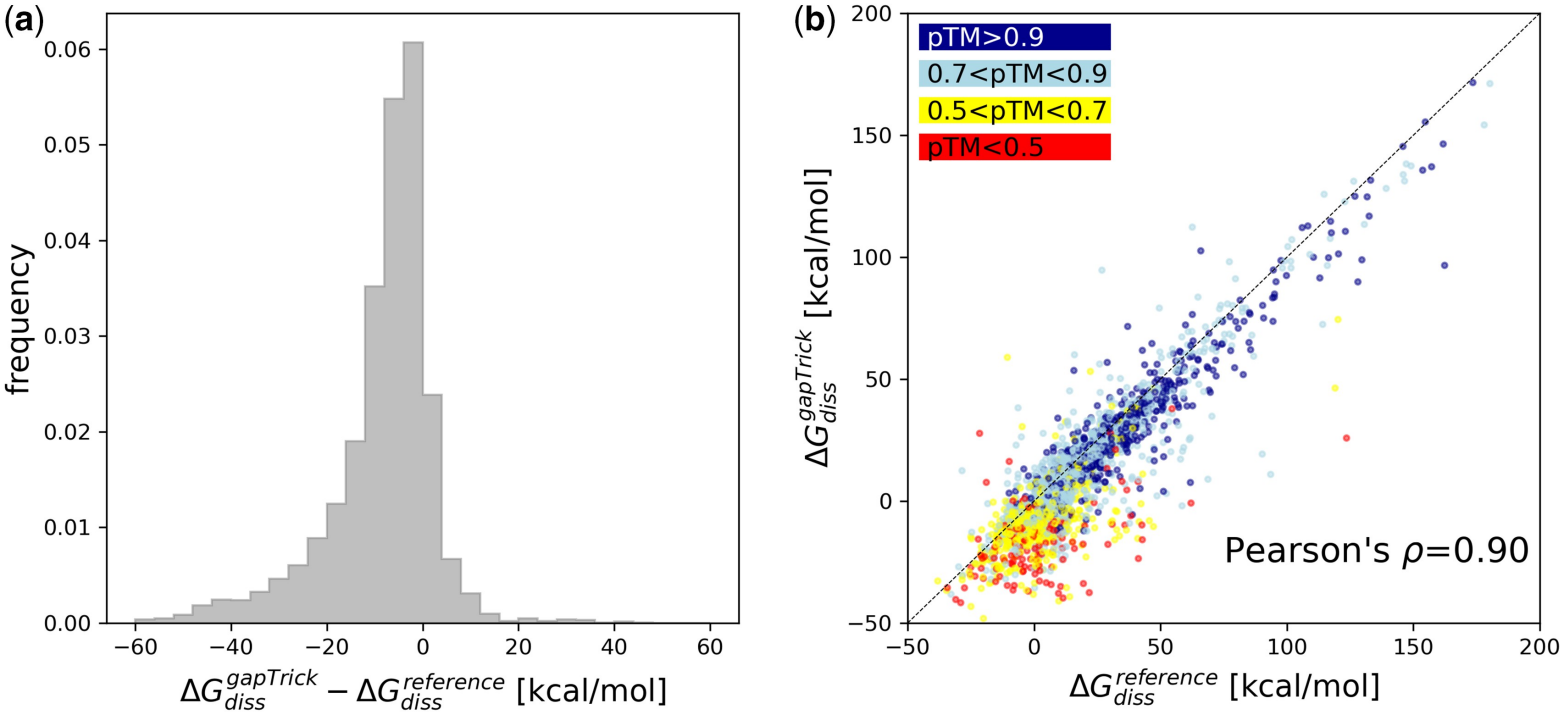
Comparison of Gibbs free energy of dissociation estimates for gapTrick predictions (Δ$G_{\text{diss}}^{\text{gapTrick}}$) and reference models (Δ$G_{\text{diss}}^{\text{reference}}$). The distribution of a difference between gapTrick and reference model Δ*G*_diss_ (a) and a scatterplot showing their strong linear correlation (b). The dashed line in panel (b) shows the diagonal of the plot and a perfect correlation of the abscissa and ordinate values. The colour code indicates the pTM score of the gapTrick predictions.

In the presented benchmarks, the Gibbs free energy of dissociation (Δ*G*_diss_) estimates for the gapTrick models are clearly lower than reference for the small fraction of the predictions (Fig. 3a). This is not surprising, as the accuracy of AF2 prediction coordinates has been estimated to be comparable with medium to low resolution crystal structures (Shao *et al.* 2022). The issue, however, affects mainly low-confidence models (Fig. 3b). Although there is a strong linear correlation between the two dissociation energy estimates (Pearson’s *r* = 0.90 ± 0.01), it is noticeably lower for medium to low-confidence predictions with pTM < 0.7 (Pearson’s *r* = 0.58 ± 0.04). There is also a small, negative linear correlation between the dissociation energies difference between predicted and reference models and pTM (Pearson’s *r*=−0.28 ± 0.02), indicating that Δ*G*_diss_ is often underestimated for low-confidence predictions.

### 3.4 Identification of correct complexes based on gapTrick contact predictions

So far, I only considered structurally characterized protein–protein complexes with good evidence that they form stable interactions. For some gapTrick applications, however, it would be very important to verify the correctness of predicted models, for example in cases where several alternative templates exist.

To test this, I created three benchmark sets consisting of AlphaFold3 predictions for pairs of proteins unlikely to form complexes (hu.MAP2 negative PPIs), likely to form complexes (hu.MAP2 positive PPIs), and models extracted from PDB-deposited cryo-EM structures resolved at a resolution worse than 4 Å.

In the absence of reference models, it is not possible to evaluate the accuracy of AF3 predictions for hu.MAP2 positive/negative PPIs. However, as the AF3 ranking scores are significantly higher in the positive test (with a *t*-test *P*-value below 0.0001), it can be assumed that at least some of the structures in the positive set are correct. Furthermore, due to AF3’s tendency to hallucinate (Abramson *et al.* 2024), it can be expected that most of the models will look plausible and have good stereochemical properties even if they are completely wrong.

**Figure 4.**
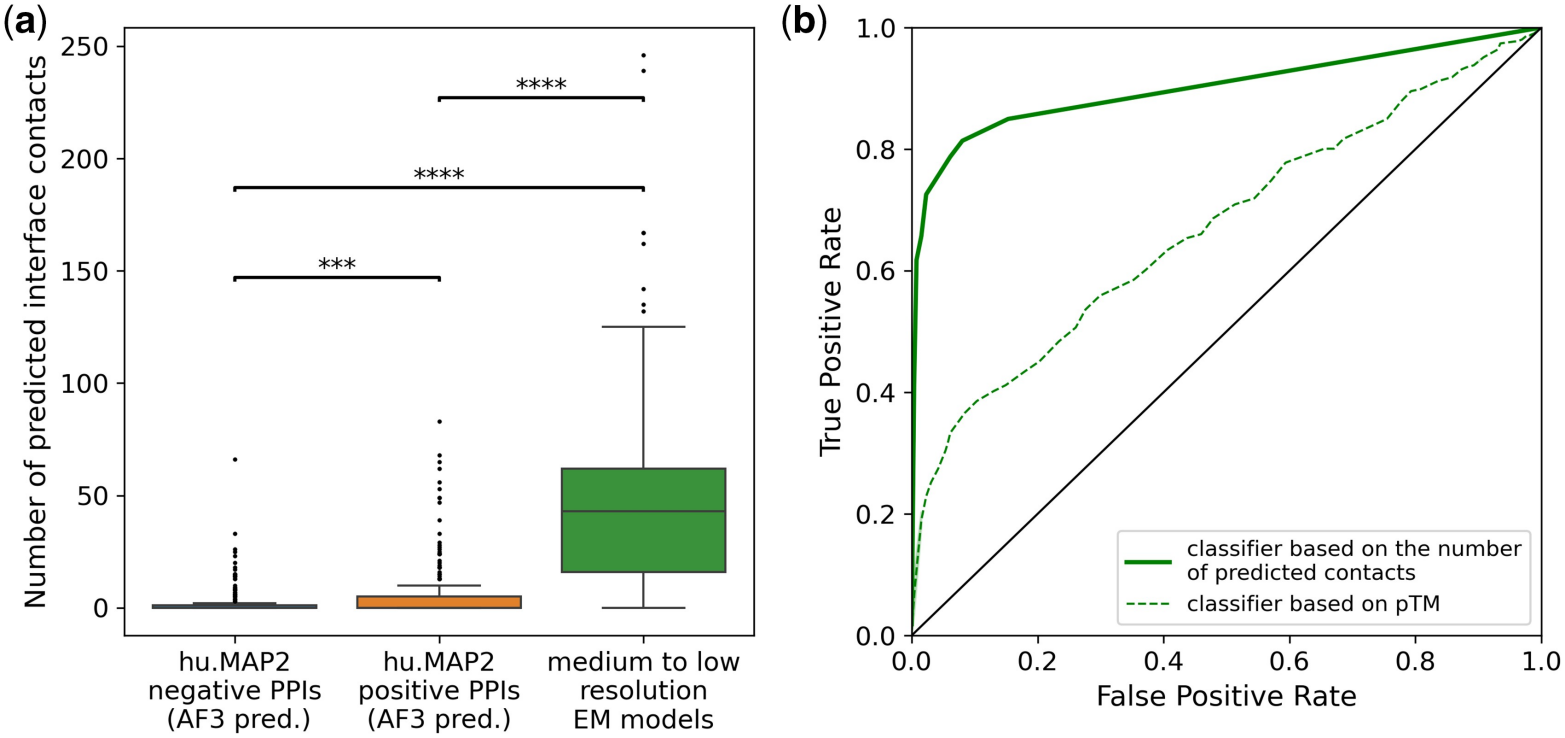
(a) A comparison of the distributions of the number of protein–protein interface contacts predicted by gapTrick. The predictions are based on templates from AF3 complex predictions for hu. Map 2.0 negative and positive PPI sets or medium-to-low resolution cryo-EM binary complexes extracted from PDB. Stars indicate the significance of two-sample *t*-tests with the hypothesis that the expectation value of a distribution on the left is smaller. (b) ROC curves of classifiers based on pTM or the number of protein–protein interface contacts predicted by gapTrick (area under the ROC curve 0.67 and 0.90).

All AF3 predictions and cryo-EM models were automatically rebuilt using gapTrick. The difference in the number of predicted protein–protein interface contacts is a very strong discriminator between the sets (Fig. 4a). It can also be used to construct a reliable classifier that discriminates between very unlikely but geometrically plausible (negative PPIs) and structurally validated cryo-EM complexes (Fig. 4b). It has much stronger predictive power than the pTM scores estimated by gapTrick (monomeric AF2 NN models do not estimate ipTM, Fig. 4b). This clearly demonstrates that the number of predicted contacts at the protein–protein interface can be used to identify plausible predictions. According to the benchmarks, models with as few as 3 predicted contacts should be correct in over 85% of cases, with a false positive rate of 16%, which corresponds to precision and recall of 86% and 85%, respectively. This is much higher than the classification performance obtained by Humphreys *et al.* (Humphreys *et al.* 2024) using monomeric AF2 NN models without templates, based on the probability of a single, most-likely inter-chain contact below 12 Å.

### 3.5 Characterization of protein–protein interactions in cryo-EM maps

**Figure 5.**
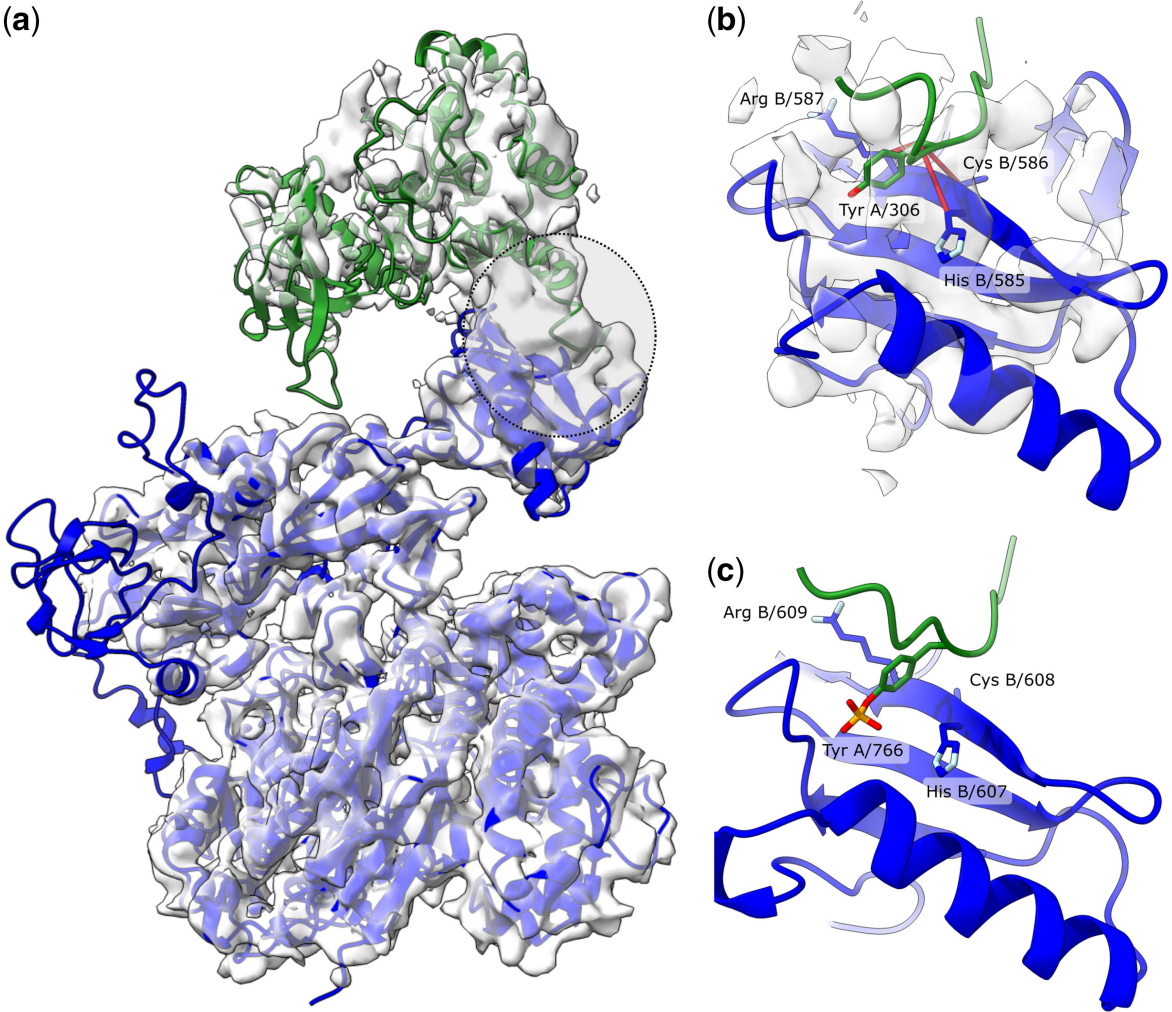
Cryo-EM structure of a human phospholipase hPLCγ2 in complex with tyrosine kinase FGFR1 resolved at 4.0 Å resolution (a). Panel (b) shows a noisy map region at the interface of the hPLCγ2-FGFR1 complex, and the corresponding fragment of the model built, fitted to the map using gapTrick. The region is highlighted with a dashed circle on panel (a). Red lines indicate high probability contact predictions between hPLCγ2 interface residues and a highly conserved Tyr766 of FGFR1. A fragment of the deposited FGFR1 model (PDB 8jqi) superposed by the displayed fragment of the hPLCγ2 (residues A/530–630, Cα rmsd 1.4 Å) is shown in black. Panel (c) shows a corresponding fragment of a 2.5 Å resolution crystal structure model of a related complex of human FGFR1 with rat rPLCγ1 (PDB 3gqi). The residues predicted by gapTrick to be in contact in the hPLCγ2-FGFR1 complex are strictly conserved in rPLCγ1 and have been identified as part of a canonical FGFR1 binding site. Phospholipases rPLCγ1 and hPLCγ2 are shown in blue, FGFR1 in green.

Phospholipase Cγ (PLCγ) enzymes control many vital cellular processes. Mutations in these enzymes can lead to abnormal cell signalling and the development of cancer. In a recent study, a complex of the human hPLCγ2 with the regulatory tyrosine kinase FGFR1 was determined using cryo-EM (Shin *et al.* 2024), but the 4.1 Å resolution of the reconstruction did not allow for a mechanistic description of the complex formation (PDEB/EMDB ids 8jqi/36573, Fig. 5a). Although a highly conserved, phosphorylated Tyr766 of FGFR1 was previously identified as the major site of interaction between FGFR1 and PLCγ2, all the structural context was available from a related crystal structure of an FGFR1 in complex with rat rPLCγ1 (PDB id 3gqi) (Bae *et al.* 2009). Here, I show that gapTrick can successfully provide the structural context of the PLCγ2–FGFR1 complex formation based on the medium-resolution cryo-EM map alone.

AlphaFold3 fails to predict the experimentally observed protein–protein interface, with and without phosphorylation of Tyr766 (ipTM/TM-score 0.35/0.49 and 0.55/0.49, respectively). Therefore, I built the model from AF2 structure predictions of individual chains [AlphaFold Protein Structure Database (Varadi *et al.* 2022) version 4 models for Uniprot P16885 and P11362]. The chains were fitted into the map using MOLREP (Vagin and Teplyakov 2010), refined in real space with self-restraints generated at the 4.3 Å cut-off in COOT (Casañal *et al.* 2020), and rebuilt using gapTrick. The resulting prediction had a pTM of 0.81 and excellent stereochemical properties (MolProbity score of 1.15). Automated refinement using Servalcat (Yamashita *et al.* 2021), with jelly body restraints and default parameters, converged in as few as 20 cycles, with no need for interactive rebuilding. The resulting model has map-model fit parameters comparable to the deposited model (CC_mask_ of 0.73 and 0.72, for deposited and rebuilt models, respectively). Although no rotamer or Ramachandran plot restraints were used during refinement, the final model has very good stereochemical properties (MolProbity score 1.23 compared to 2.25 for the deposited model).

The prediction includes several high probability contacts at the hPLCγ2-FGFR1 interface (Fig. 5b). The contacts correspond to the previously identified canonical binding site of the phosphorylated Tyr766 of FGFR1 and closely resemble a previous crystal structure model of human FGFR1 with a rat phospholipase rPLCγ1 (Fig. 5c) (Bae *et al.* 2009). The Gibbs free energy of dissociation estimated for the new model is −1.5 kcal/mol and comparable to the deposited hPLCγ2-FGFR1model (0.5 kcal/mol) and the corresponding rPLCγ2-FGFR1 crystal structure (1.8 kcal/mol). In light of the results presented in this paper, this relatively weak complex would be difficult to predict, regardless of the well-studied and biologically relevant interaction mechanism.

### 3.6 Comparison between gapTrick and AF_unmasked

The use of multimeric templates was deliberately disabled by the AF2 developers. However, a recent publication has shown that the software can be modified to allow the use of inter-chain contact information from templates to improve prediction performance with AF_unmasked (Mirabello *et al.* 2024). In addition to an artificially generated benchmark set, the AF_unmasked method was tested on a set of 28 heterodimeric protein complexes that cannot be predicted by AlphaFold2 (DockQ < 0.2), but for which a homologous complex is available in the PDB (DockQ > 0.15). The authors showed that most of the test set structures can be correctly predicted based on the homologous templates.

**Figure 6.**
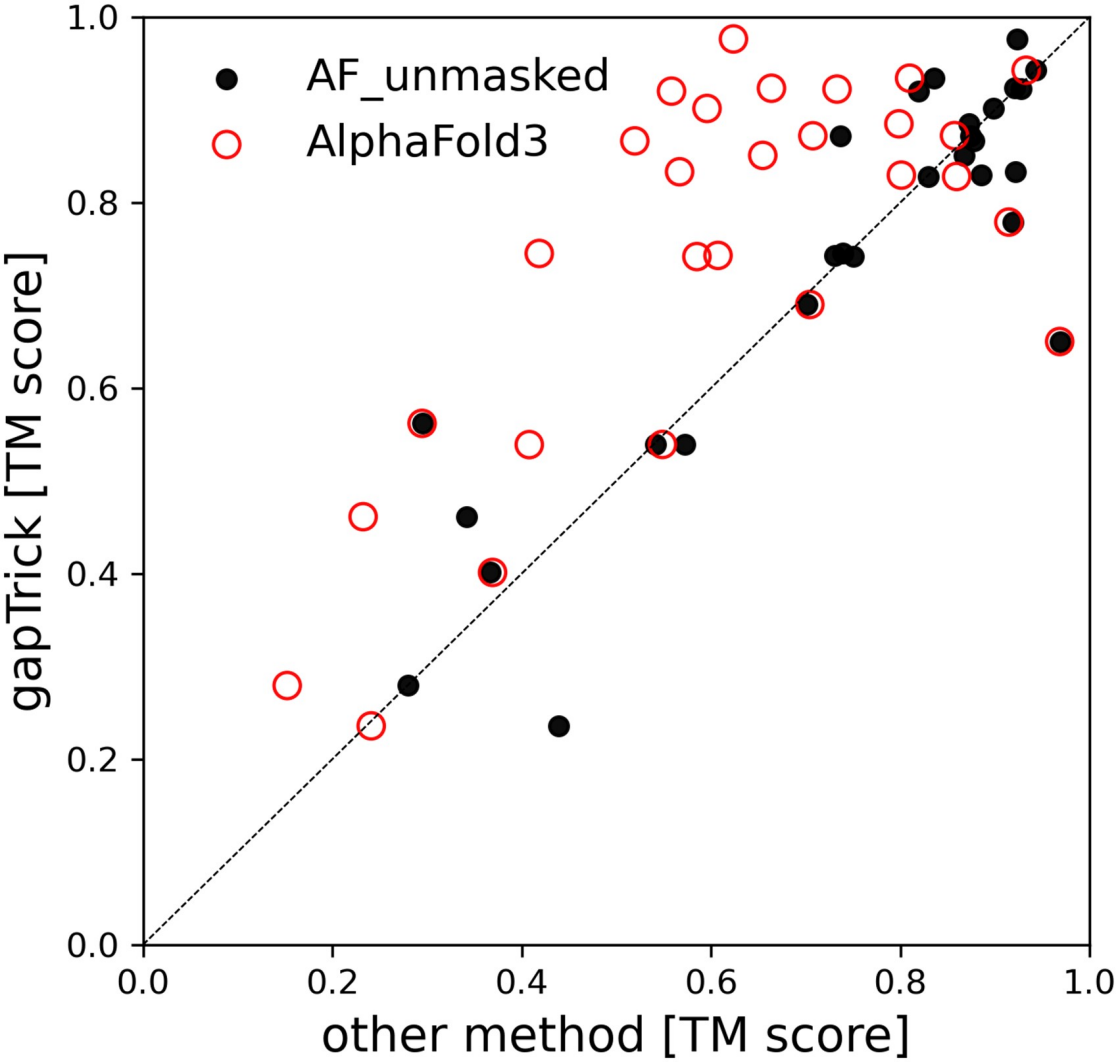
Comparison of the performance of gapTrick, AF_unmasked, and AlphaFold3 on a set of 28 heterodimeric proteins defined by Mirabello *et al.* (2024). Both gapTrick and AF_unmasked predictions were generated using complexes homologous to the target as templates.

Here, I compared the performances of gapTrick and AF_unmasked on this test set. For comparison, I used predicted structures provided by the AF_unmasked authors to avoid any issues related to a non-optimal configuration of their software. The gapTrick predictions were generated fully automatically using default parameters. The model accuracies (TM-score against a reference) of the two approaches are highly correlated (Fig. 6). They are also significantly better than the AF3 predictions, although it is not clear whether the set of heterodimeric complexes and the AF3 training set share any homologous structures that might bias the predictions.

A more comprehensive comparison of gapTrick and AF_unmasked is beyond the scope of this work due to the problems with defining the benchmark strategy, which is much more difficult for the latter. Most importantly, it is not trivial to avoid information leakage between training and test sets for a model trained on protein complexes (Bernett *et al.* 2024). Furthermore, unlike gapTrick, AF_unmasked requires user-defined mapping between template chains and target sequences and is not applicable for automated, large-scale processing of higher oligomers. Finally, only gapTrick provides tools for the analysis of predicted contacts.

## 4 Discussion

I show that AlphaFold3 often fails to predict structures of weak complexes, which may be transient or depend on the presence of additional components that are not included in the prediction (Fig. 1, available as supplementary data at *Bioinformatics* online). The prediction of such complexes is crucial for the identification of novel PPIs for which a complete composition, required for a stable complex formation, has not yet been identified. Modelling of small fragments of protein complexes can also be important for building very large cryo-EM models (Mosalaganti *et al.* 2022). Therefore, it is very important to include all known interaction partners in the prediction, including other proteins, cofactors, and substrates. In some cases, the full composition and stoichiometry of protein complexes—information necessary for identifying interactions between their components—can be inferred computationally using AlphaFold-Multimer-based approaches (Chim and Elofsson 2024, Shor and Schneidman-Duhovny 2024, Molodenskiy *et al.* 2025). However, such methods are computationally expensive and often yield ambiguous results that require careful interpretation.

Many weak complexes can be predicted using gapTrick when a low-accuracy multimeric template is already available. The templates can be homologous structures or rigid-body fits of the complex components to a cryo-EM map using approaches like Slice’N’Dice (Simpkin *et al.* 2025). Templates can also originate from *de novo* cryo-EM map interpretation approaches such as ModelAngelo (Jamali *et al.* 2024), which typically produce fragmented and incomplete models in regions of lower local resolution. In such cases, gapTrick can support tedious and error-prone model building using interactive methods. It can also complement automated approaches such as PDB_REDO (Joosten *et al.* 2014) or CERES (Liebschner *et al.* 2021). Particularly important here are the excellent stereochemical properties of models predicted by gapTrick, as detailed in Fig. 2, available as supplementary data at *Bioinformatics* online. The model geometry should not deviate from energetically favourable conformations unless there is very good evidence in the data supporting unusual conformations (Wlodawer 2017).

Models of protein complexes, like any other scientific model, require experimental validation. This usually involves structure-driven identification of interactions that are critical for its function *in vivo*. I have demonstrated that gapTrick can identify crucial contacts in low-accuracy models of protein complexes whose overall structures cannot be accurately predicted using standard approaches. These contacts are predicted with high precision and can help to assess model correctness and to define residues for mutagenesis in functional analysis. They are also a strong indicator of model correctness, which will be very important in cases where there is no other evidence of complex formation and topology.

The availability of suitable multimeric templates is a key limitation of gapTrick. The benchmarks presented in this work are based on low-accuracy models assembled from target chains. This reflects a scenario in which gapTrick is used, e.g. for cryo-EM model building where initial models are assembled from individual target predictions (as demonstrated with the PLCγ2–FGFR1 complex). When the template is incomplete or derived from a homologous structure, particular care must be taken to ensure that sequence alignment between the target and template is accurate, as poor alignment can hinder the effective use of template information.

## Supplementary Material

btaf532_Supplementary_Data

## Data Availability

The gapTrick source code is maintained at https://github.com/gchojnowski/gapTrick. The code version used for obtaining results presented here was archived at https://doi.org/10.5281/zenodo.17038359.

## References

[btaf532-B1] Abramson J , Adler J, Dunger J et al Accurate structure prediction of biomolecular interactions with AlphaFold 3. Nature 2024;630:493–500. 38718835 10.1038/s41586-024-07487-w PMC11168924

[btaf532-B2] Agirre J , Atanasova M, Bagdonas H et al The CCP4 suite: integrative software for macromolecular crystallography. Acta Crystallogr D Struct Biol 2023;79:449–61. 37259835 10.1107/S2059798323003595 PMC10233625

[btaf532-B3] Bae JH , Lew ED, Yuzawa S et al The selectivity of receptor tyrosine kinase signaling is controlled by a secondary SH2 domain binding site. Cell 2009;138:514–24. 19665973 10.1016/j.cell.2009.05.028 PMC4764080

[btaf532-B4] Bernett J , Blumenthal DB, List M. Cracking the black box of deep sequence-based protein—protein interaction prediction. Brief Bioinform 2024;25:bbae076. 38446741 10.1093/bib/bbae076 PMC10939362

[btaf532-B5] Bryant P , Pozzati G, Elofsson A. Improved prediction of protein-protein interactions using AlphaFold2. Nat Commun 2022;13:1265. 35273146 10.1038/s41467-022-28865-w PMC8913741

[btaf532-B6] Burke DF , Bryant P, Barrio-Hernandez I et al Towards a structurally resolved human protein interaction network. Nat Struct Mol Biol 2023;30:216–25. 36690744 10.1038/s41594-022-00910-8 PMC9935395

[btaf532-B7] Burnley T , Palmer CM, Winn M. Recent developments in the CCP-EM software suite. Acta Crystallogr D Struct Biol 2017;73:469–77. 28580908 10.1107/S2059798317007859 PMC5458488

[btaf532-B8] Casañal A , Lohkamp B, Emsley P. Current developments in Coot for macromolecular model building of Electron Cryo‐Microscopy and Crystallographic Data. Protein Sci 2020;29:1069–78. 31730249 10.1002/pro.3791 PMC7096722

[btaf532-B9] Chim HY , Elofsson A. MoLPC2: improved prediction of large protein complex structures and stoichiometry using Monte Carlo Tree Search and AlphaFold2. Bioinformatics 2024;40:btae329. 38781500 10.1093/bioinformatics/btae329 PMC11194477

[btaf532-B10] Chojnowski G , Sobolev E, Heuser P et al The accuracy of protein models automatically built into cryo-EM maps with ARP/wARP. Acta Crystallogr D Struct Biol 2021;77:142–50. 33559604 10.1107/S2059798320016332 PMC7869898

[btaf532-B11] Cock PJA , Antao T, Chang JT et al Biopython: freely available Python tools for computational molecular biology and bioinformatics. Bioinformatics 2009;25:1422–3. 19304878 10.1093/bioinformatics/btp163 PMC2682512

[btaf532-B12] Drew K , Wallingford JB, Marcotte EM. hu.MAP 2.0: integration of over 15,000 proteomic experiments builds a global compendium of human multiprotein assemblies. Mol Syst Biol 2021;17:e10016. 33973408 10.15252/msb.202010016 PMC8111494

[btaf532-B13] Evans R , O’Neill M, Pritzel A et al Protein complex prediction with AlphaFold-Multimer. bioRxiv, 2021:2021–2010, preprint: not peer reviewed.

[btaf532-B14] Gao M , Nakajima An D, Parks JM et al AF2Complex predicts direct physical interactions in multimeric proteins with deep learning. Nat Commun 2022;13:1744. 35365655 10.1038/s41467-022-29394-2 PMC8975832

[btaf532-B15] Grosse-Kunstleve RW , Sauter NK, Moriarty NW et al The Computational Crystallography Toolbox: crystallographic algorithms in a reusable software framework. J Appl Crystallogr 2002;35:126–36.

[btaf532-B16] Humphreys IR , Zhang J, Baek M et al Protein interactions in human pathogens revealed through deep learning. Nat Microbiol 2024;9:2642–52. 39294458 10.1038/s41564-024-01791-x PMC11445079

[btaf532-B17] Hunter JD. Matplotlib: a 2D graphics environment. Comput Sci Eng 2007;9:90–5.

[btaf532-B18] Jamali K , Käll L, Zhang R et al Automated model building and protein identification in cryo-EM maps. Nature 2024;628:450–7. 38408488 10.1038/s41586-024-07215-4 PMC11006616

[btaf532-B19] Joosten RP , Long F, Murshudov GN et al The PDB_REDO server for macromolecular structure model optimization. IUCrJ 2014;1:213–20. 10.1107/S2052252514009324 PMC4107921 25075342

[btaf532-B20] Jumper J , Evans R, Pritzel A et al Highly accurate protein structure prediction with AlphaFold. Nature 2021;596:583–9. 34265844 10.1038/s41586-021-03819-2 PMC8371605

[btaf532-B21] Kosinski J , von Appen A, Ori A et al Xlink analyzer: software for analysis and visualization of cross-linking data in the context of three-dimensional structures. J Struct Biol 2015;189:177–83. 25661704 10.1016/j.jsb.2015.01.014 PMC4359615

[btaf532-B22] Krissinel E. Crystal contacts as nature’s docking solutions. J Comput Chem 2010;31:133–43. 19421996 10.1002/jcc.21303

[btaf532-B23] Krissinel E , Henrick K. Inference of macromolecular assemblies from crystalline state. J Mol Biol 2007;372:774–97. 17681537 10.1016/j.jmb.2007.05.022

[btaf532-B24] Liebschner D , Afonine PV, Moriarty NW et al CERES: a cryo-EM re-refinement system for continuous improvement of deposited models. Acta Crystallogr D Struct Biol 2021;77:48–61. 33404525 10.1107/S2059798320015879 PMC7787109

[btaf532-B25] Mirabello C , Wallner B, Nystedt B et al Unmasking AlphaFold to integrate experiments and predictions in multimeric complexes. Nat Commun 2024;15:8724. 39379372 10.1038/s41467-024-52951-w PMC11461844

[btaf532-B26] Mirdita M , Schütze K, Moriwaki Y et al ColabFold: making protein folding accessible to all. Nat Methods 2022;19:679–82. 35637307 10.1038/s41592-022-01488-1 PMC9184281

[btaf532-B27] Molodenskiy D , Maurer VJ, Yu D et al AlphaPulldown2—a general pipeline for high-throughput structural modeling. Bioinformatics 2025;41:btaf115. 40088942 10.1093/bioinformatics/btaf115 PMC11937959

[btaf532-B28] Mosalaganti S , Obarska-Kosinska A, Siggel M et al AI-based structure prediction empowers integrative structural analysis of human nuclear pores. Science 2022;376:eabm9506. 35679397 10.1126/science.abm9506

[btaf532-B29] Oliphant TE. A Guide to NumPy. USA: Trelgol Publishing USA, 2006.

[btaf532-B30] Pettersen EF , Goddard TD, Huang CC et al UCSF ChimeraX: structure visualization for researchers, educators, and developers. Protein Sci 2021;30:70–82. 32881101 10.1002/pro.3943 PMC7737788

[btaf532-B31] Poweleit N , Czudnochowski N, Nakagawa R et al The structure of the endogenous ESX-3 secretion system. Elife 2019;8:e52983. 31886769 10.7554/eLife.52983 PMC6986878

[btaf532-B32] Prisant MG , Williams CJ, Chen VB et al New tools in MolProbity validation: caBLAM for CryoEM backbone, UnDowser to rethink “waters,” and NGL viewer to recapture online 3D graphics. Protein Sci 2020;29:315–29. 31724275 10.1002/pro.3786 PMC6933861

[btaf532-B33] Roney JP , Ovchinnikov S. State-of-the-art estimation of protein model accuracy using AlphaFold. Phys Rev Lett 2022;129:238101. 36563190 10.1103/PhysRevLett.129.238101 PMC12178128

[btaf532-B34] Sánchez Rodríguez F , Simpkin AJ, Chojnowski G et al Using deep-learning predictions reveals a large number of register errors in PDB depositions. IUCrJ 2024;11:938–50. 10.1107/S2052252524009114 PMC11533997 39387575

[btaf532-B35] Shao C , Bittrich S, Wang S et al Assessing PDB macromolecular crystal structure confidence at the individual amino acid residue level. Structure 2022;30:1385–94.e3. 36049478 10.1016/j.str.2022.08.004 PMC9547844

[btaf532-B36] Shin Y-C , Plummer-Medeiros AM, Mungenast A et al The crystal and cryo-EM structures of PLCγ2 reveal dynamic interdomain recognitions in autoinhibition. Sci Adv 2024;10:eadn6037. 39612343 10.1126/sciadv.adn6037 PMC11606444

[btaf532-B37] Shor B , Schneidman-Duhovny D. CombFold: predicting structures of large protein assemblies using a combinatorial assembly algorithm and AlphaFold2. Nat Methods 2024;21:477–87. 38326495 10.1038/s41592-024-02174-0 PMC10927564

[btaf532-B38] Simpkin AJ , Elliot LG, Joseph AP et al Slice’N’Dice: maximising the value of predicted models for structural biologists. Acta Crystallogr D Struct Biol 2025;81:105–21. 39976565 10.1107/S2059798325001251 PMC11883665

[btaf532-B39] Sobolev OV , Afonine PV, Moriarty NW et al A global Ramachandran score identifies protein structures with unlikely stereochemistry. Structure 2020;28:1249–58 e1242. 32857966 10.1016/j.str.2020.08.005 PMC7642142

[btaf532-B40] Steinegger M , Söding J. MMseqs2 enables sensitive protein sequence searching for the analysis of massive data sets. Nat Biotechnol 2017;35:1026–8. 29035372 10.1038/nbt.3988

[btaf532-B41] The wwPDB Consortium. Protein Data Bank: the single global archive for 3D macromolecular structure data. Nucleic Acids Res 2019;47:D520–8. 30357364 10.1093/nar/gky949 PMC6324056

[btaf532-B42] Tokuriki N , Tawfik DS. Stability effects of mutations and protein evolvability. Curr Opin Struct Biol 2009;19:596–604. 19765975 10.1016/j.sbi.2009.08.003

[btaf532-B43] Tunyasuvunakool K , Adler J, Wu Z et al Highly accurate protein structure prediction for the human proteome. Nature 2021;596:590–6. 34293799 10.1038/s41586-021-03828-1 PMC8387240

[btaf532-B20677778] Turner J, , Abbott S, , Fonseca N et al EMDB—the electron microscopy data bank. Nucleic Acids Res 2024;52:D456–65. 10.1093/nar/gkad1019 37994703 PMC10767987

[btaf532-B44] Turner J , Abbott S, Fonseca N et al; The wwPDB Consortium. EMDB—the Electron Microscopy Data Bank. Nucleic Acids Res 2024;52:D456–D465. 37994703 10.1093/nar/gkad1019 PMC10767987

[btaf532-B45] Vagin A , Teplyakov A. Molecular replacement with MOLREP. Acta Crystallogr D Biol Crystallogr 2010;66:22–5. 20057045 10.1107/S0907444909042589

[btaf532-B46] van Kempen M , Kim SS, Tumescheit C et al Fast and accurate protein structure search with Foldseek. Nat Biotechnol 2024;42:243–6. 37156916 10.1038/s41587-023-01773-0 PMC10869269

[btaf532-B47] Varadi M , Anyango S, Deshpande M et al AlphaFold protein structure database: massively expanding the structural coverage of protein-sequence space with high-accuracy models. Nucleic Acids Res 2022;50:D439–D444. 34791371 10.1093/nar/gkab1061 PMC8728224

[btaf532-B48] Virtanen P , Gommers R, Oliphant TE et al; SciPy 1.0 Contributors. SciPy 1.0: fundamental algorithms for scientific computing in Python. Nat Methods 2020;17:261–72. 32015543 10.1038/s41592-019-0686-2 PMC7056644

[btaf532-B49] Wlodawer A. Stereochemistry and validation of macromolecular structures. Protein Crystallogr Methods Protoc 2017;1607:595–610. 10.1007/978-1-4939-7000-1_24 PMC5560084 28573590

[btaf532-B50] Wojdyr M. GEMMI: a library for structural biology. J Open Source Softw 2022;7:4200.

[btaf532-B51] Wukovitz SW , Yeates TO. Why protein crystals favour some space-groups over others. Nat Struct Biol 1995;2:1062–7. 8846217 10.1038/nsb1295-1062

[btaf532-B52] Yamashita K , Palmer CM, Burnley T et al Cryo-EM single-particle structure refinement and map calculation using Servalcat. Acta Crystallogr D Struct Biol 2021;77:1282–91. 34605431 10.1107/S2059798321009475 PMC8489229

