# Supplementary material for: gapTrick—structural characterization of protein–protein interactions using AlphaFold
Source: Bioinformatics. 2025 Sep 23;41(9):btaf532. doi: 10.1093/bioinformatics/btaf532 (PMC12479393; doi:10.1093/bioinformatics/btaf532)
Supplement: btaf532_Supplementary_Data [file btaf532_supplementary_data.pdf]

gapTrick – Structural characterisation of protein-protein interactions using AlphaFold

Grzegorz Chojnowski

European Molecular Biology Laboratory, Hamburg Unit, Notkestrasse 85, 22607, Hamburg, Germany.

1. Weak interfaces can be predicted in the context of fully assembled complexes

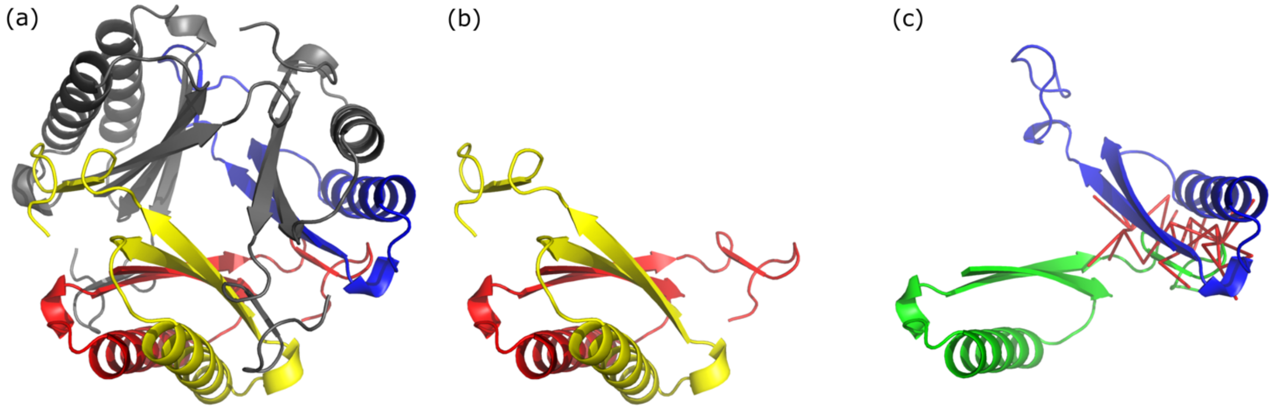

Figure S1 An AlphaFold3 prediction of the hexametric structure of 4-oxalocrotonate tautomerase from *Pseudomonas putida* (a). The model has very high prediction confidence scores pTM/iPTM=0.94/0.94 This structure contains a larger dimeric interface between two subunits (b), which can be predicted in isolation using AlphaFold3, again with high confidence scores (pTM/ipTM=0.85/0.86). Prediction of a smaller dimeric interface in isolation can only be pursued with gapTrick based on a template (c). The template-based prediction reveals multiple high-probability interactions, which is strong evidence of the model correctness (pTM=0.83).

2. Stereochemical properties of models rebuilt with gapTrick

A convenient application of gapTrick is rebuilding and completion of models fitted into cryo-EM or MX maps. It can be particularly useful when a high-confidence prediction of a complete complex is not available and low map resolution doesn't allow *de novo* model tracing. In such cases, the complex models must be assembled in the maps from individual chains, which doesn't allow for detailed modelling of PPI interfaces. I have shown that gapTrick can successfully remodel such structures and predict contacts that may be crucial for assessing their correctness. However, the overall plausibility of the stereochemical properties of such models remains an open question.

The stereochemical properties of macromolecules have been studied in detail over the years using both theoretical and statistical approaches to define energetically favourable conformations. The model geometry should not deviate from these values unless there is very good evidence in the data supporting an unusual conformation, which is usually not the case at lower resolutions (Wlodawer, 2017). Therefore, reliable stereochemical properties of gapTrick predictions are crucial, as they facilitate refinement, model interpretation, and subsequent deposition to the PDB.

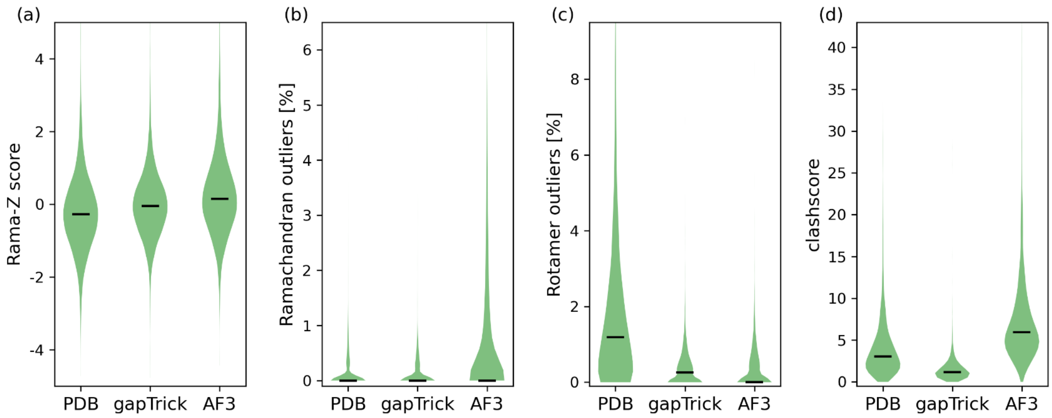

Figure S2 Comparison of the most important MolProbity validation scores estimated for models predicted using gapTrick, AlphaFold3 (AF3), and PDB-deposited models. To reduce bias due to the presence of experimentally unresolved, and often intrinsically disordered regions, the predicted models were truncated to residue ranges covered by PDB-deposited structures prior to scoring. Horizontal lines on the violin plots represent medians.

The models predicted by gapTrick have stereochemical properties reported by MolProbity (Prisant, et al., 2020) that are comparable to or better than those of the PDB-deposited crystal structure models and AlphaFold3 predictions (Figure S2). They have reliable distributions of backbone torsion angles (Figure S2a), with only 1% significantly different from those observed for high quality crystal structures (absolute value of Rama-Z score above 3)(Sobolev, et al., 2020). The corresponding fractions for PDB-deposited and AF3-predicted models from the benchmark set are 2% and 3%, respectively. The gapTrick predictions have a comparable number of Ramachandran plot outliers to the fully refined crystal structure models (Figure S2b). They have very few non-rotameric side chains, which is very important for the interpretation of medium and low-resolution maps (Figure S2c). Finally, the gapTrick models have a very low clashscore (number of steric conflicts per 1000 atoms in the model) with a median of 1.2 (Figure S2d). This is significantly lower than the median clashscore of the input templates, with randomly shifted and rotated chains, which is as high as 44. These results clearly show that gapTrick automatically resolves most of the stereochemical issues in the templates, and that they do not require additional pre-processing and rebuilding.

References

Prisant, M.G., *et al.* New tools in MolProbity validation: CaBLAM for CryoEM backbone, UnDowser to rethink "waters," and NGL Viewer to recapture online 3D graphics. *Protein Sci* 2020;29(1):315-329.

Sobolev, O.V., *et al.* A Global Ramachandran Score Identifies Protein Structures with Unlikely Stereochemistry. *Structure* 2020;28(11):1249-1258 e1242.

Wlodawer, A. Stereochemistry and validation of macromolecular structures. *Protein Crystallography: Methods and Protocols* 2017:595--610.
